# Public-School Outrages

**Published:** 1868-01

**Authors:** 


### HALL'S JOURNAL OF HEALTH.

Our Legitimate Scope is almost boundless; for whatever begets pleasurable and harmless feelings, promotes Health; and whatever induces disagreeable sensations, engenders Disease.

WE AIM TO SHOW HOW DISEASE MAY BE AVOIDED, AND THAT IT IS BEST, WHEN SICKNESS COMES, TO TAKE NO MEDICINE WITHOUT CONSULTING A PHYSICIAN.

VOL. XV.] JANUARY, 1868. [No. 1.

### PUBLIC-SCHOOL OUTRAGES.

If every Public-School building in the city of New York were razeed to its nethermost foundation-stone, and every school-commissioner and school trustee were tarred-and-feathered, then ridden on forty rails and then hung up by the big toe and drenched with ice-water until they had learned the most infinitesimal mite of common-sense, decency and humanity, ten thousand parents would raise their pæans high, and a quarter of a million of children would shout their glad songs of deep thanksgiving.

Some time ago a remark was made in a public vehicle that a certain public building had been burned down. "I wish it had been the Twelfth Street public-school," replied a young girl of some fifteen years, who had a good arm-load of school-books which she was carrying to the hateful place.

These may seem harsh, hard and hasty words, but we personly know fathers and mothers in different parts of the city, who execrate the whole system, and cannot speak of it without a feeling of the deepest indignation at the inexcusable outrages which are practiced every day at these much lauded establishments, but whose scanty means in these times of high rents, high prices and high taxes, compel them to send their children to these schools or let them grow up in ignorance. We have been repeatedly and earnestly urged for years past, by intelligent fathers and mothers to draw public attention to these matters, but it was an ungracious subject, and the thing was passed over, but it is meet that with the New Year, a new leaf should be turned over—with what effect time only can tell; but time will do it, by the aid of the increasing intelligence of the people.

Multitudes are driven to patronize the public schools, because it is impossible for them pecuniarily, to give their daughters the blessing—stop, reader! we prefer another word; the curse of an education in the Female Boarding and Day Schools of this metropolis, which, with possibly some very few exceptions, are the hot-beds of vicious practices, wrecking soul, body and estate with their wicked, withering and destructive influences. It would be worse than useless to write thus, without specifications terse, truthful and to the point.

We know a gentleman who paid eight hundred dollars a year for the education of an orphan neice in one of these private female schools. We know a lady who sent two daughters from near Albany to one of these establishments, and the bills were sixteen hundred dollars a year; "extras," all told.

Look at the programme of any of these establishments, which pretend to give the finishing touch to a young lady's education, and nearly every branch, not to be learned at a common good country school, is charged "extra." What they do learn is, for a very great part, a mere feat of memory. A girl who had been at one of these, was sent to a public school, and on being asked what was the shape of the world replied with great promptness, and yet with some caution, "It's square, I suppose." Another who was made ashamed by the apparent greater advancement of those around her said, "If I had been at the Public School I should have known something by this time."

The bills for books and stationery alone at the private schools amount to one or two hundred dollars; if these lady teachers are not hired in some way to use these books, they can say so. No two schools use the same text-books; and not half or quarter of the books are mastered, and nothing like it; some of them not even read, unless in the most cursory way.

One of the features in these private pandemoniums is the culture of dress; no sooner is one examination or soiree or seance over than there begins to be a talk about when the next one is to come off, and what kind of a new dress must be had; and as each daughter is a favorite or pet at home, and loved so tenderly, parents do not like to have their feelings hurt or their pride wounded by being behind the foremost in the matter of personal adornment, and thus the first lessons are given in those extravagances which are to beggar future husbands, and land hoards of destitute orphans at the Five Point Missions, or fill the streets with ragged newsboys and the public asylums with diseased paupers. These pestiferous establishments fit young ladies to "appear well in society," so as to marry rich husbands, and adorn the drawing-room; but they do not fit them to become the wives of steady, ambitious and thrifty young men, who have their fortunes to make, and such are far more desirable for husbands, are better matches than the sons of millionaires who, in too many cases, grow up into manhood without mastering any calling, profession or trade; and who, if they lose their inheritance, would have to be supported by their wives. If there is a single female boarding-school in all New York which educates its girls to be good wives, good house-keepers and good mothers, such an one has not been heard of, up to the present date of writing.

If these things are so, then does the charge well lay, that there is not in all the city of New-York a boarding and day school worthy of the patronage of thoughtful and provident parents ; hence, they all merit extinction, and their proprie- tors, as far as the public good is concerned, ought to have a ticket of leave to follow some more honest occupation.

But we sat out to prove that the public school nuisance should be abated, or placed upon a better foundation. We have said nothing in this article against the superintendents and teachers of the public schools ; on the contrary, we have written for years in high commendation of them, and they were commendations well deserved ; as a class they merit the respect of this whole community for their assiduity, for their patience, for their tact and general intelligence. It is the public school system which we are anathemizing and the authors and founders of the same, in the persons of school commissioners and school trustees ; these are they who make the rules, laws and regulations; and the teachers are com- pelled to carry them out or lose their places. These men were formerly grocery keepers, shoulder-hitters, prize fighters and bullies, and "ignorami" in general; not every one, but the majority ; some, within our own knowledge, have been taken up, while in office, for theft, burglaries, assaults and batteries and attempted murders ; not every one of them certainly, for now and then a decent man has been elected ; but they were in such a tremendous minority that they could do nothing. Within a year, a better system of selection for the manage- ment of the public schools has been inaugurated, and no doubt there are some worthy men who hold the office of school commissioners and school trustees, but their hands are tied or they are dumb prophets, while their intelligence and respectability only give credit to an office which is a disgrace to civilization. And now for some facts, but while learning them, let it be under the supposition that you are reading the history of your own dear daughter, your own favorite son, tortured to death by slow degrees and now resting in the peaceful grave.

Go to any public school in New-York, where are congregated from one to two thousand children; let them be all summoned to the large room; ask the teachers who are the best scholars in each class, and put down the name, age and residence. Take another book, and go along each bench, and take down the name, age and residence of the runtiest, thinnest, palest, scabbiest scholars; compare the books, and the names will be coincident with a most appalling uniformity. A good sized, robust, healthful looking boy or girl, who stands at the head of the class or any where near head, will be a curiosity for Barnum's Museum. It must not be forgotten that some children are exceedingly perverse and that to manage such wisely and well, is much more than many parents can do, and requires on the part of the faithful teacher a patience worthy of Job and a wisdom not a whit less than that of Solomon, and that the public school teachers succeed so generally in keeping their classes up to the studying point, without parental enforcement at home, deserves great praise.

It must be remembered also, that the teachers are expected to pass over a certain ground of study and that if they do not bring the children forward, they are liable to lose their living, and in the effort to do this and yet not oppress the children with lessons to be learned at home is next to an impossibility and some errors of judgment are unavoidable and ought to be laid to the charge of the trustees and commissioners.

We have known most unreasonable tasks to be given out on Fridays for Monday's recitations, and when mute murmers have been manifested in the involuntary exclamations, or visible deep frowns of the passive victims, the reason for such procedure has been extorted, "you have a good long holiday between this and Monday to learn them in," while at the same time it has been inculcated that it is a sin to study the lessons on Sundays. The inevitable result is, the scholar must violate its tender conscience, must study all day Saturday or have the lash of "failure" inflicted, a sentence more terrible to a sensitive young child than a cat-o-nine-tails: this whole thing is simply horrible.

In order to save time for study, many children ride in the cars or stages to school, so that while running they may read and any observer can note their sad and anxious faces of any morning; faces which ought to be lighted up with radiance and gladness. We know a girl of seventeen who goes to bed at eleven o'clock and long before day is sitting up in her bed with a shawl around her shoulders and by a glaring gaslight, is as much bent on her studies as if it were a matter of life and death.

We know a girl preparing for graduation who is so oppressed by a desire to keep up with her class, that under the continued influence of anxiety, and late hours and want of sleep seldom eats any breakfast, and at five o'clock in the afternoon sits down to the first and only real meal of the twenty-four hours Nothing short of a miracle or some most extraordinary counteracting influence can save that girl from being a life-long dyspeptic to grieve her parents, to worry her husband, and under the irritating influence of disease, to poison all the vitals to fret and harass and scold her children and servants from morning until night, and from one month's end to another; nor does it end here; the maladies of mind and body are perpetuated to children's children filling society in the coming age with the embodiment of moral, mental and physical disease. A happier fate has fallen to some. I was called to see Miss W., the only daughter of a retired millionaire of a New England city — she was eighteen years of age Had just graduated with the first honors. She had been so excited for several months previous, so fully bent on carrying the palm, that she seemed to have no appetite for food; the mind appeared to feed on the body; she would often eat while going to school and study while thus eating and walking along; the parents saw her declining every day; but they knew her object, sympathised with her ambitions, hoped that the school would soon be over and that then they would hasten away in travel and retrieve her health, but the day after she graduaded she was taken ill, and when first seen was hopelessly ill, and soon after died.

Miss S. C. was a beautiful black eyed girl, with a temper so sweet, a heart so loving, and a disposition so gentle, so dutiful, so conscientious, that her parents looked upon her as the pride of their life now, and to be the stay and comfort of their age. She was fourteen; the announcement was made that there would be an examination for promotion, and the failure of promotion is more to a school girl than a sentence to the penitentiary to a Wall Street broker, whose body and soul are so rhinocerosed as to feel nothing short of an earthquake. How hard she tried to be prepared for the examination, with ali her nice sensibilities, it is painful even to think of. Her very sensitiveness caused her failure. She said nothing and came home with a settled sadness on her features; no cheerful word passed her lips; no smile lighted up her countenance, no merry twinkle as of yore played about her keen black eyes which looked so lovingly in their depth. She ate nothing and moved about the house like an automaton; to every tender inquiry from a tender mother's heart "is any thing the matter with my dear daughter?" the most unsatisfactory of all answers would be listlessly given, "nothing." Yet both parents felt satisfied that mortified pride was eating out her life. The school authorities were appealed to, to reverse their decision, as a means of probably saving her life, especially as the "failure" was most likely a fault of memory only; as it was known that she was one of the most obedient, orderly and attentive students of the whole school. The reply was "guess she will get over it, the rules of the school can't be changed." In a few days more she was confined to her bed, typhoid symptoms manifested themselves; in the mutterings of delirium parts of the hateful lessons were repeated and she died; one of the sweetest girls that ever blessed a parent's love: murdered outright by injudiciously exacted studies.

Miss B. wanted to keep up with her class; she was not bright, but she was beautiful, with a family name of which any New-Yorker might well be proud. She sat up late; she rose early; she gave no time for recreation; she ate irregularly; slept imperfectly; neglected every thing but her studies; Saturday and Sunday were all alike dreadful task-days to her, but it was no use; she could not keep up: the mind was gone and she died demented.

Within six months Miss J., accompanied by her parents, applied for medical advice. She seemed so young, so confiding, so dutiful and so good, it was impossible not to feel a deep sympathy for her; she had just completed her studies; her ambition had led her to over application for the preceeding two years; but the stamina of life was all gone and she must die within the year. These are all cases personally known to us; they are plain statements of facts just as they occurred in public and private schools, and in the light of them, the thoughtful reader will probably join in the sentiments expressed in the opening of this article.

A single fact is very suggestive in another direction. We know a boy of thirteen, a lively lad, who stands well in his classes, has missed none of the promotions, but he is a runt; not as tall as his younger sister by nearly one foot; he has so little sleep during the week, that on Saturdays and Sundays he sleeps until ten, eleven and even twelve o'clock; without these makings up, involving loss of Sunday school and church, he could not live healthfully as to body or mind. Sometimes a whole class is "kept in" after having already been in the tread mill of study from nine until three, because all seemed to have committed some mischief; now it is perfectly certain that all were not guilty, thus the guilty and innocent are punished alike; such a gross injustice must injure the moral sense of any conscientious child. We have known lessons to be given out to be learned between school-hours, so extravagantly disproportioned to the time and capacity of the children that out of a class of fifty or sixty not more than two or three could recite the lesson acceptably; then half the lesson was given with an unsatisfactory result; and a part of that half was given for the third morning, showing a criminal want of judgment.

In the delicate matter relating to the calls of nature inexcusable barbarities are sometimes practised; there are several "intermissions" during school hours for this and other reasons, yet it will happen in certain cases of sickness or forgetfulness, that these are not sufficient, and the rule is to stand up or hold the hand up until the teacher notices it, which may not be in half an hour or an hour or not at all; children have been known to faint in the school room, under such disagreeable and dangerous and always hurtful circumstances; and parents have been compelled before now to remove their children from the school on this account. A wreck is often made of the consciences of children in the public school in many little ways. All know how tender the mind is of children whose mothers try to bring them up to all that is truthful and right. Speaking to a child next you is forbidden; that child may worry or tease, and if getting out of all patience the other becomes restless or speaks out, punishment is inflicted without inquiring minutely into the circumstances.

It is forbidden to look on the slate or book of another scholar; conscientious children will not do this; most don't hesitate to do it, and by this aid are "promoted" while the good child is disgraced by not using these advantages.

In very many cases, parents who have to work for a living have to spend hours of valuable time in aiding their little ones to "get their lessons." Many children have to work when they get home in order to help their parents through with their work and having to get their lessons besides, the hardship of their position is apparent, and in the struggle to get along, health is often lost and a life-time of suffering is the result. The remedies proposed for some of these enormities are:

First, no child under fifteen years of age ought to be kept in school over four hours in any twenty-four.

Second, no child under fifteen years of age ought to be allowed to take any book out of the school-room.

Third, no examinations ought to be allowed for any purpose. or on any pretext whatever.

Fourth, all promotions and all diplomas should be given on the basis of general good conduct and efficiency.

'ifth, no man ought to be accessible to the office of school-management, unless he has actually taught school seven years.

This thing of school examinations is an absurdity, a farce and a sham; they are not intended for the benefit of the scholar, but the glorification of the teacher. A scholar who passes an examination is not a whit better scholar an hour after than he was the hour before; and a competent teacher ought to know as well before an examination as after, whether a scholar should pass or not.

It is well known that many a scholar passes by a trick, or by a mere feat of memory, or sheer impudence; while a conscientious child, better qualified, fails to pass from diffidence or want of memory

The great idea in school teaching should be to make the study agreeable, this alone would show the capability and genius of the teacher

The second important point is, to master first principles, in other words, to be thorough, thorough, thorough. A child should not be allowed to make a single advance in any study until what has gone before has been wholly understood and made level to the capacity of the child; it is the neglect of these two things on the part of the teacher which has driven multitudes of children from school and caused a failure of an education which might have made them successful men and women; and in stead of that, they have "run off," have gone to sea or have made of themselves common vagabonds. Make learning pleasurable, be thorough, this is the way to make children love study and to make good scholars, the world over, and in all ages and nations, and without these the school house will be a purgatory and education a hateful thing. Four hours in school, four hours in the fields every day, rain or shine, these are first things.

The following just remarks are from the New-York Observer, under the head of

CHILDREN ARE ANIMALS.

Every day wnen we come down town we meet children going to school with their arms full of books. In the afternoon we meet them going home from school with the same load. These books they are to study at their own homes. After dinner they are to sit down, and work away at fractions, or French exercises, grammar or geometry, something to worry their weary brains with till it is time for them to go to bed. There they are to think of what they have been studying, and when they rise in the morning, the lessons must be looked over, and kept in mind till they are recited in school.

This is all wrong. We have a society to prevent cruelty to animals, and we wish Mr. Berg would attend to this matter. Is not a child of more value than many turtles? If the Society is justly exercised over the condition of distressed calves, has it not bowels of mercies for the poor rich children of the fashionable seminaries and institutes and schools for young ladies up town? No child ought to be allowed to study one moment out of school hours. These hours my include three in the forenoon and three in the afternoon, though five in a day are better than six. All the rest of the twenty-four should be given to exercise, recreation, sleep and social duties and pleasures. The child is an animal, not a machine, not a block or a stone. Its nervous organization is delicate, easily deranged, and in school-days peculiarly sensitive and exposed to injury. Parents do not think of it, and teachers do not regard it, but the neglect is cruel and wicked, and the child suffers a sad penalty for the sin of others.

Will some one tell us of a first class schoor where a child may be placed and positively prohibited by its teachers from studying out of the regular hours of school? Who manage the Public Schools? The New-York Tribune of Nov. 26, 1867, answers that question in the following words:

One of the "rig'lar Dimmycratic" nominees for School Officer in an up-town Ward rejoices in the name of Michael

Ryan, for short called Mike Rine. This robust Irishman has great fame in his locality, and expects (with good reason) to be returned to the local Board, so that he can regulate Dave Scott and other merely native subordinates who teach and tremble under his smiles and frowns. There are few great men whose histories do not furnish anecdotes illustrative of their peculiar character and views. Mr. Ryan is no exception. For instance: Some years ago Mr. Curtis (we presume most intelligent men will remember him) was a School Commissioner and President of the Board of Education. During his term Mr. Ryan was a member to the Eighteenth Ward Democratic Nomination Convention. When the Convention met, some of the light-colored sort of Democrats proposed to nominate Curtis for reelection, as he was the most efficient and one of the most able of all members of the Board. Thereupon, up arose the Honorable Mr. Ryan, and "objikted" "to the nominishun of Misther Curtis." Being pressed for his reasons for this extraordinary objection to a regular Democrat, the illustrious Ryan stated that Curtis was "too much of a "gintleman." An honorable member of that Convention, since dead, was so much disturbed by this exhibition that he wasted much breath in rebuking the great Ryan, but his words fell upon the impervious object as harmlessly as rain-drops upon the pavements.

The reader may feel somewhat comforted in the fact that New-York city is not singular in its propensity for the selection of ragamuffins and know-nothings for school-managers; for it seems that a school trustee in West Virginia, who could neither read nor write, was obliged to take a census of the children in his district. He accomplished it by filling a pocket with red and white beans. When he met a boy he put a red bean in a side pocket, and girls were represented by white beans in another receptacle. When he thought he had got all, he counted the beans.

### MEMORIES RUINED.

The general tendency of the manner in which the public schools are conducted in New-York city is to make study a mere mechanical action of the memory and then destroy it.

The ambition of the child is to recite his lesson correctly and by whatever means, fair or foul, he can escape the terrible sentence of "failure" he will adopt; he will cheat, prevaricate, and lie remorselessly; they do it every day; nine out of ten of all of them will do it; and that the terror the sentence of "failure" should drive to these things is a moral enormity which ought to be abated, in our opinion, even if every school house was burned down, and every trustee in it.

So great is the desire to "say the lesson" acceptably that the whole effort and being of the child is expended in the direction of impressing the lesson on the memory and after the lesson is said there is a reaction on the mind and body of the child, which makes a single thought about the past lesson a burden, and it is instinctively shunned and the result is in very many cases, that the next day the lesson is in a measure forgotten. A single illustration in our own hearing is sufficient; four children had been going to the public school; the younger, five years, in coming to a lesson it seemed a little hard, and each of the other three children of greater age were applied to, and every one answered "I have forgotten it." Whereas if the principle had ever been impressed on the mind, it could not have been forgotten; similar exhibitions as to the fact of past studies having escaped the memory have been noticed in a great multitude of cases.

The tyranny which the dread of bad marks and failures exercises on the mind of the children of the public schools is not merely severe; it is terrible; but we see no hope of a change until the parents become wise enough to care at least as much for the wellfare of their children at school as for their canary birds, horses, cats and poodle dogs.

